# Association of Triglyceride-Glucose Index With In-Hospital Malignant Arrhythmias in Older Patients With Diabetes Mellitus and Left Ventricular Aneurysm: A Retrospective Study

**DOI:** 10.31083/RCM47761

**Published:** 2026-05-18

**Authors:** Jinghao Zhou, Qian Lu, Jijie Jin, Binyan Chen, Shengzhang Chen, Jianghua Zhou, Shanzhen Shi

**Affiliations:** ^1^School of Nursing, Wenzhou Medical University, 325000 Wenzhou, Zhejiang, China; ^2^Department of Cardiology, The First Affiliated Hospital of Wenzhou Medical University, 325000 Wenzhou, Zhejiang, China

**Keywords:** left ventricular aneurysm, triglyceride–glucose index, malignant arrhythmias, insulin resistance

## Abstract

**Background:**

Left ventricular aneurysm (LVA), a severe post-myocardial infarction (MI) complication, predisposes patients to malignant arrhythmias. The triglyceride-glucose (TyG) index, a marker of insulin resistance, is linked to adverse cardiac outcomes. Nevertheless, the association between the TyG index and in-hospital malignant arrhythmias in older, diabetic patients with LVA remains unclear.

**Methods:**

This retrospective study (the First Affiliated Hospital of Wenzhou Medical University, China) consecutively enrolled 655 patients with LVAs (aged ≥60 years with confirmed diabetes) from 2004 to 2024. The relationship between the TyG index quartiles and in-hospital malignant arrhythmias was evaluated using Kaplan-Meier (K-M) curves, Cox regression, and restricted cubic spline (RCS) analyses. Mediation analysis was employed to investigate underlying inflammatory pathways.

**Results:**

Participants had a median age of 75 years, and 57.56% were males. The RCS analysis indicated that elevated TyG index values were associated with a higher risk of in-hospital malignant arrhythmias; compared with the lowest quartile (Q1), the highest quartile (Q4) showed a nearly threefold higher risk (hazard ratio [HR] = 2.99; 95% confidence interval [CI]: 1.26–7.13). Exploratory pathway analyses indicated that neutrophils (NEs), white blood cells (WBCs), and the systemic inflammation response index (SIRI) partially accounted for this association. Subgroup analyses revealed no significant interactions.

**Conclusions:**

This study shows that the TyG index is independently associated with in-hospital malignant arrhythmias in older patients with LVA, serving as a simple, clinically relevant marker to identify high-risk individuals and guide targeted management.

## 1. Introduction

Left ventricular aneurysm (LVA) is one of the serious complications after acute myocardial infarction (MI), with an incidence of 5%–15% [1, 2, 3]. LVA occurs predominantly after an anterior wall myocardial infarction and results from pathological left ventricular remodeling, during which necrotic myocardium is replaced by fibrotic scar tissue, leading to ventricular dilation, structural deformity, and abnormalities in electrical conduction [4, 5, 6]. Consequently, patients with LVA are at an increased risk for malignant arrhythmias, thrombus formation, heart failure, and sudden cardiac death despite timely reperfusion therapy.

In recent years, the triglyceride-glucose (TyG) index, calculated from fasting triglyceride and glucose levels, has been widely recognized as a reliable and economical surrogate for insulin resistance (IR) [7]. Accumulating evidence has associated elevated TyG levels with multiple cardiovascular conditions, including coronary atherosclerosis, adverse postoperative cardiovascular events, and increased mortality in chronic heart failure, suggesting its potential value in cardiovascular risk assessment [8, 9].

Older adults who develop LVA after MI represent an especially vulnerable population [10]. Age-related myocardial fibrosis, impaired autonomic regulation, and a high prevalence of co-morbidity collectively increase the risk of electrical instability, making malignant arrhythmias more frequent and more hazardous in elderly individuals with LVA [11, 12]. However, whether the TyG index can be used to stratify risk for in-hospital malignant arrhythmias in this population remains unknown. Therefore, by investigating the relationship between the TyG index and in-hospital malignant arrhythmias in elderly individuals with LVA, this study aims to provide an accessible and economical tool for early risk stratification.

## 2. Materials and Methods

### 2.1 Study Design and Participants

**Fig. 1.**
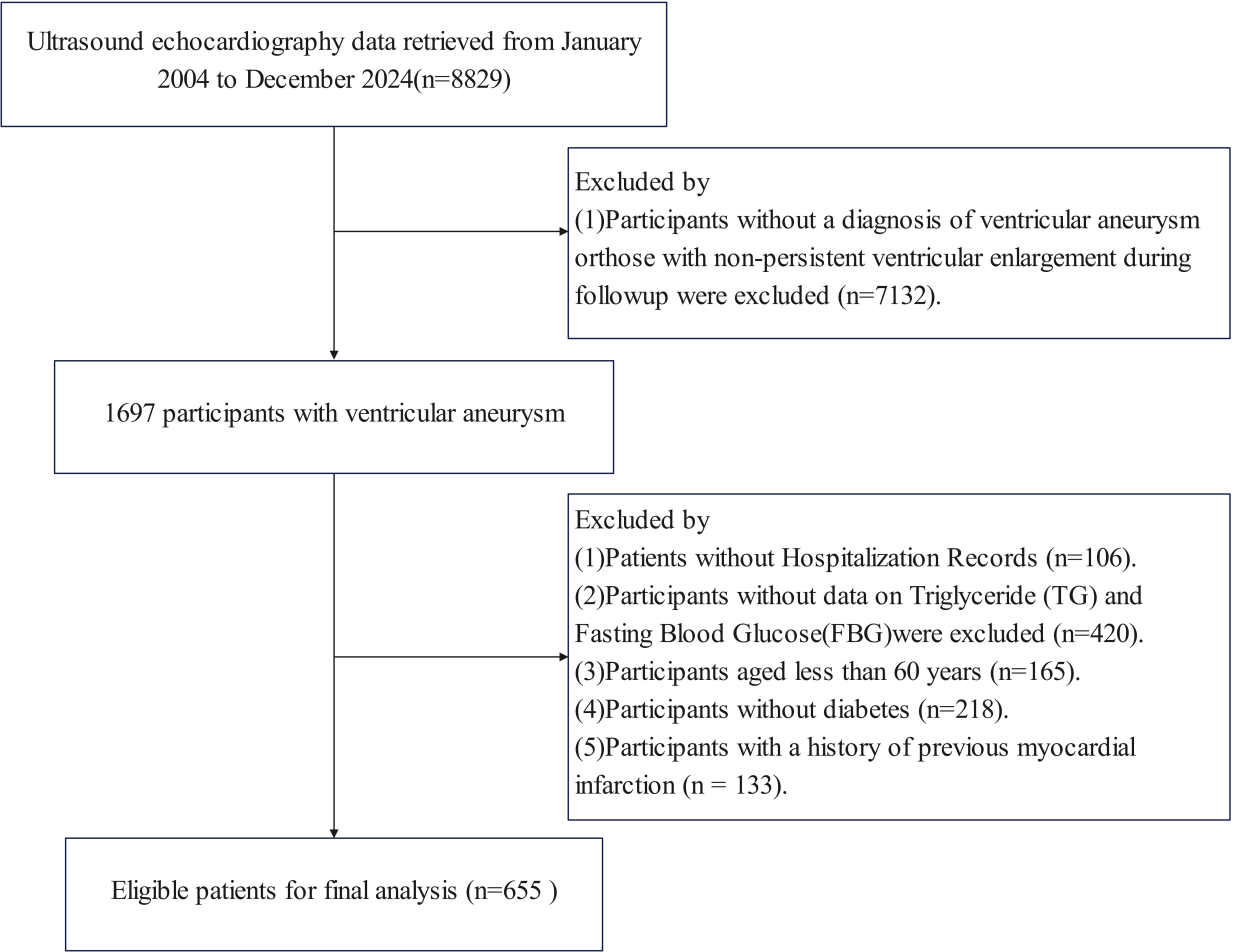
**Flowchart for subject screening and study design**.

This retrospective observational cohort study included 655 patients diagnosed with ventricular aneurysm (VA) at the First Affiliated Hospital of Wenzhou Medical University from January 2004 to December 2024. An outline of the screening workflow is illustrated in Fig. 1. Ultrasound echocardiography data were retrieved from January 2004 to December 2024 (n = 8829). Participants without a diagnosis of ventricular aneurysm or those with non-persistent ventricular enlargement during follow-up were excluded (n = 7132), leaving 1697 participants with ventricular aneurysm. Further exclusions included: (1) patients without hospitalization records (n = 106); (2) participants for whom triglyceride (TG) and fasting blood glucose (FBG) data were unavailable (n = 420) (these missing records were from earlier years of the dataset); (3) participants aged less than 60 years (n = 165); (4) participants without diabetes (n = 218); (5) participants have a history of previous MI (n = 133). This resulted in 655 eligible patients with first-ever acute MI complicated by new-onset LVA for the final analysis.

### 2.2 Data Collection

Patient clinical information was collected from prior hospital records and included demographics (age, sex, and body mass index [BMI] (BMI was dichotomized at 24 kg/m^2^ according to the World Health Organization (WHO) Asia-Pacific criteria and the Chinese adult overweight guidelines, which define BMI ≥24 kg/m^2^ as overweight [13])); clinical risk factors (blood pressure indices [systolic blood pressure (SBP), diastolic blood pressure (DBP)], lifestyle factors such as smoking and alcohol consumption, and medical history of diabetes mellitus, hypertension, and hyperlipidemia (the latter defined based on prior clinical diagnosis supplemented by baseline lipid profiles: total cholesterol (TC) >5.2 mmol/L, TG >1.7 mmol/L, low-density lipoprotein cholesterol (LDL-C) >3.4 mmol/L, or high-density lipoprotein cholesterol (HDL-C) <1.0 mmol/L [14])). and pharmacotherapy-related covariates included the use of lipid-lowering drugs, angiotensin receptor blockers (ARBs), angiotensin-converting enzyme inhibitors (ACEIs), beta-blockers, aldosterone antagonists, antiarrhythmic agents, and antidiabetic medications prior to admission, as well as the in-hospital use of beta-blockers, aldosterone antagonists, and antiarrhythmic drugs. In addition, the analysis included two percutaneous coronary intervention (PCI) variables: PCI status (No PCI, Emergency, Elective) and PCI type (Conservative, Stent, Balloon, or combinations).

Laboratory indices, including TC, TG, LDL-C, HDL-C, glycosylated hemoglobin (HbA1c), FBG, estimated glomerular filtration rate (eGFR), white blood cell (WBC) count, B-type natriuretic peptide (BNP), c reactive protein (CRP), monocytes count, lymphocyte count and neutrophil (NE) count , were obtained from routine clinical testing performed during hospitalization. All measurements adhered to standardized protocols and testing systems.

Echocardiographic data were collected from routine examinations performed during hospitalization. Given the long study span, two objectively verifiable data sources were available. For earlier cases, measurements were extracted directly from archived echocardiography reports. For more recent cases with preserved digital records, original DICOM images were retrieved and independently reviewed by two experienced echocardiographers following a standardized interpretation protocol. The echocardiographic data included left ventricular end-systolic dimension (LVESD), left ventricular end-diastolic dimension (LVEDD), and left ventricular ejection fraction (LVEF).

### 2.3 Study Definitions

The study diagnosed a VA using the transthoracic echocardiography (TTE). The diagnostic criteria were: persistent outward bulging of the left ventricular wall during the cardiac cycle, characterized by thinning of the ventricular wall, absence of contraction, or paradoxical motion, and a clear demarcation from adjacent normal myocardium [15]. Diagnosis of diabetes mellitus followed the American Diabetes Association (ADA) standards, including a documented diagnosis of diabetes in the electronic medical record, treatment with insulin or oral antidiabetic agents, and laboratory findings of FBG ≥126 mg/dL or HbA1c ≥6.5% [16].

### 2.4 Calculation of TyG

The TyG index quantifies insulin IR by combining fasting glucose with triglyceride levels. It was calculated as Ln (fasting TG [mg/dL] × FBG [mg/dL] / 2) [17]. The participants were classified into four groups (Q1, Q2, Q3, Q4) by the quartiles of the TyG index, with Q1 serving as the reference group. Using the fully adjusted model, receiver operating characteristic (ROC) curve analysis was performed to determine the optimal cutoff value for discrimination.

### 2.5 Follow-Up and Endpoint Events

Follow-up data in this study were collected from medical records of both inpatients and outpatients, with the follow-up period calculated from the date of VA diagnosis until discharge or death, whichever occurred first. The main outcome assessed in this study was the occurrence of in-hospital malignant arrhythmias, which specifically included sudden cardiac death and episodes of ventricular tachycardia (VT) or ventricular fibrillation (VF). Sudden cardiac death was defined as sudden cardiac arrest due to cardiac causes in previously stable individuals, excluding non-cardiac causes such as trauma, poisoning, or cerebrovascular events. Ventricular tachycardia or ventricular fibrillation was defined as episodes requiring treatment with an implantable cardioverter-defibrillator (ICD) or untreated episodes of ventricular tachycardia lasting longer than 30 seconds detected by an ICD. The diagnosis was based on electrocardiogram (ECG) or ICD recordings [18].

### 2.6 Statistical Analysis

In this study, continuous data were summarized as mean ± standard deviation (SD) or median (interquartile range, IQR) depending on normality. Differences were evaluated using one-way analysis of variance (ANOVA) for normally distributed variables and the Kruskal-Wallis test for non-normal data. Categorical variables, reported as frequencies (percentages), were compared via the chi-square test or Fisher’s exact test.

To comprehensively evaluate the association between the TyG index and in-hospital malignant arrhythmias, this metric was analyzed in three complementary ways: (1) as a continuous variable for Cox regression and restricted cubic spline (RCS) analyses to characterize the overall dose-response relationship; (2) as quartiles to illustrate risk gradients across TyG levels using Kaplan-Meier curves; (3) as a binary variable, using an optimal cutoff determined by ROC analysis (derived from the multivariate model) to assess its potential clinical applicability. Cox regression models were performed to estimate hazard ratios (HRs) and 95% confidence intervals (CIs). Three models were constructed: Model 1 was unadjusted; Model 2 adjusted for age, gender, BMI, smoking, and alcohol consumption; Model 3 additionally incorporated blood parameters, medication use, and echocardiographic variables. Kaplan-Meier curves with log-rank tests were used to compare event incidence across TyG quartiles and across ROC-derived high- and low-TyG groups. RCS analysis was applied to assess potential nonlinear associations between continuous TyG levels and clinical outcomes.

Inflammatory markers were considered as potential biomarkers mediating the relationship between the TyG index and in-hospital malignant arrhythmias [19]. Exploratory pathway analyses using structural equation modelling (SEM) were conducted to examine whether inflammatory markers could statistically account for the link between the TyG index and in-hospital malignant arrhythmias. Exploratory single-pathway models were first constructed for NE and WBC to estimate the proportion of the association they explained. A multiple-pathway model was then fitted to evaluate their independent and combined contributions, considering potential overlap between these inflammatory measures. The statistical significance of these pathways was assessed using 5000 bootstrap resamples to obtain robust estimates. Additionally, the Systemic Inflammation Response Index (SIRI) was also evaluated for its potential to statistically explain this association. Subgroup analyses were conducted across key demographic, clinical, metabolic, lifestyle, and treatment-related categories, with interaction effects evaluated using the likelihood ratio test. Variance inflation factor (VIF) analysis revealed that values for all covariates were less than 5, suggesting the absence of substantial multicollinearity (**Supplementary Table 1)**. Statistical computations were carried out via R software (version 4.5.1; R Foundation for Statistical Computing, Vienna, Austria), defining statistical significance as a two-tailed *p*-value < 0.05.

## 3. Result

### 3.1 Baseline Characteristics of Study Participants

Table 1 presents the baseline characteristics of the 655 participants according to TyG index quartiles. The cohort had a mean age of 75 years, with 57.56% being male. The average TyG index was 8.82 (8.42–9.33). Compared with the lowest quartile group, participants with the highest quartile group tended to be younger, female, obese, and non-smokers, and had a higher prevalence of hyperlipidemia and hypertension, along with greater use of beta-blockers during hospitalization. The biochemical parameters showed significant differences across the groups. Significantly increased DBP, HbA1c, LDL-C, CRP, WBC, monocyte count and NE were observed in the highest quartile. ROC curve analysis identified 9.01 as the optimal TyG index threshold for predicting in-hospital malignant arrhythmias (**Supplementary Fig. 1**). By using this cutoff value to stratify the TyG index, **Supplementary Table 2** presents the baseline characteristics of the patients. The baseline characteristics of both the high TyG group and the low TyG group were largely consistent with the results of the quartile stratification.

**Table 1.** **The main baseline characteristics of eligible patients according to TyG quartiles**.

Variables		Overall	Q1	Q2	Q3	Q4	*p* value
TyG		8.82 (8.42–9.33)	8.12 (7.88–8.27)	8.60 (8.52–8.72)	9.06 (8.94–9.18)	9.62 (9.46–10.01)	<0.001
Age, years		75.00 (68.00–79.00)	76.00 (71.00–82.00)	76.00 (70.00–80.00)	73.00 (69.00–79.00)	72.00 (66.00–78.00)	<0.001
Males, n (%)		377 (57.56%)	119 (75.80%)	104 (61.90%)	77 (49.36%)	77 (44.25%)	<0.001
BMI, kg/m^2^		23.44 (21.51–25.65)	22.49 (20.54–25.28)	23.70 (21.80–25.73)	23.38 (21.48–25.16)	24.56 (22.47–26.01)	<0.001
Smoke, n (%)		226 (34.50%)	73 (46.50%)	63 (37.50%)	48 (30.77%)	42 (24.14%)	<0.001
Alcohol consumption, n (%)		114 (17.40%)	39 (24.84%)	27 (16.07%)	17 (10.90%)	31 (17.82%)	0.012
SBP (mmHg)		115.00 (103.00–130.00)	111.00 (102.00–123.00)	114.00 (105.00–130.00)	115.00 (102.00–132.00)	118.00 (105.00–134.00)	0.019
DBP (mmHg)		68.00 (61.00–75.00)	65.00 (60.00–71.00)	68.00 (62.00–75.00)	66.50 (60.00–74.25)	70.00 (61.00–78.00)	0.005
eGFR, mL/min/1.73 m^2^		66.40 (45.40–85.90)	68.90 (49.90–84.40)	70.05 (52.95–84.83)	66.75 (44.83–85.68)	62.45 (37.78–87.48)	0.109
HbA1c (%)		7.30 (6.40–8.40)	7.00 (6.20–8.00)	7.10 (6.30–8.03)	7.10 (6.40–8.20)	7.90 (7.00–9.40)	<0.001
BNP (mmol/L)		852.00 (384.00–1850.50)	803.00 (360.00–1440.00)	819.50 (291.75–1861.25)	865.00 (415.75–1862.75)	989.00 (526.00–1974.75)	0.309
TC (mmol/L)		2.72 (0.74–4.27)	2.87 (0.63–3.83)	2.74 (0.67–4.25)	2.40 (0.79–4.52)	2.77 (0.90–4.59)	0.245
TG (mmol/L)		1.22 (0.92–1.69)	0.80 (0.66–0.95)	1.12 (0.96–1.29)	1.43 (1.12–1.74)	1.93 (1.47–2.47)	<0.001
LDL-C (mol/L)		2.59 (2.05–3.23)	2.40 (1.93–2.83)	2.52 (1.90–3.10)	2.72 (2.09–3.28)	2.78 (2.18–3.69)	<0.001
HDL-C (mmol/L)		0.98 (0.80–1.17)	1.04 (0.84–1.20)	0.94 (0.77–1.14)	0.97 (0.82–1.13)	0.95 (0.79–1.17)	0.069
CRP (mg/L)		26.20 (9.90–65.55)	22.10 (8.20–66.00)	24.50 (8.63–53.93)	26.45 (9.35–56.98)	37.55 (14.7–76.25)	<0.001
WBC (×10^9^/L)		8.37 (6.44–11.13)	7.27 (5.52–9.26)	8.11 (6.36–9.96)	8.27 (6.6–10.74)	10.37 (7.74–12.99)	<0.001
NE (×10^9^/L)		6.00 (4.09–8.88)	5.00 (3.40–6.80)	5.60 (4.06–7.80)	5.75 (4.36–8.41)	8.16 (5.46–11.28)	<0.001
Lymphocyte (×10^9^/L)		1.40 (1.02–1.80)	1.28 (1.00–1.63)	1.42 (1.04–1.82)	1.40 (1.06–1.80)	1.41 (1.07–1.95)	0.061
Monocytes (×10^9^/L)		0.60 (0.43–0.88)	0.56 (0.40–0.80)	0.60 (0.40–0.81)	0.60 (0.46–0.82)	0.70 (0.48–1.06)	0.024
Hyperlipidemia, n (%)		515 (78.63%)	94 (59.87%)	134 (79.76%)	131 (83.97%)	156 (89.66%)	<0.001
Hypertension, n (%)		395 (60.31%)	78 (49.68%)	93 (55.36%)	96 (61.54%)	128 (73.56%)	<0.001
ACEI/ARB, n (%)		295 (45.04%)	79 (50.32%)	75 (44.64%)	72 (46.15%)	69 (39.66%)	0.273
Lipid lowering drug, n (%)		74 (11.30%)	21 (13.38%)	21 (12.50%)	12 (7.69%)	20 (11.49%)	0.400
Antidiabetic medication, n (%)		224 (34.20%)	24 (15.29%)	47 (27.98%)	62 (39.74%)	91 (52.30%)	<0.001
Beta-blocker, n (%)		47 (7.18%)	14 (8.92%)	17 (10.12%)	4 (2.56%)	12 (6.90%)	0.048
In-hospital beta-blocker use, n (%)		375 (57.25%)	74 (47.13%)	97 (57.74%)	98 (62.82%)	106 (60.92%)	0.023
Aldosterone antagonist, n (%)		34 (5.19%)	10 (6.37%)	9 (5.36%)	6 (3.85%)	9 (5.17%)	0.795
In-hospital aldosterone antagonist use, n (%)		365 (55.73%)	82 (52.23%)	90 (53.57%)	93 (59.62%)	100 (57.47%)	0.519
Antiarrhythmic drugs, n (%)		28 (4.27%)	10 (6.37%)	7 (4.17%)	3 (1.92%)	8 (4.60%)	0.279
In-hospital antiarrhythmic drug use, n (%)		79 (12.06%)	19 (12.10%)	21 (12.50%)	19 (12.18%)	20 (11.49%)	0.994
PCI status, n (%)							0.514
	No PCI	96 (14.66%)	24 (15.29%)	24 (14.29%)	20 (12.82%)	28 (16.09%)	
	Emergency PCI	232 (35.42%)	62 (39.49%)	53 (31.55%)	51 (32.69%)	66 (37.93%)	
	Elective PCI	327 (49.92%)	71 (45.22%)	91 (54.17%)	85 (54.49%)	80 (45.98%)	
PCI type, n (%)							0.684
	No PCI	96 (14.66%)	24 (15.29%)	24 (14.29%)	20 (12.82%)	28 (16.09%)	
	Conservative strategy	110 (16.79%)	26 (16.56%)	30 (17.86%)	22 (14.10%)	32 (18.39%)	
	Stent only	201 (30.69%)	50 (31.85%)	57 (33.93%)	50 (32.05%)	44 (25.29%)	
	Balloon only	35 (5.34%)	5 (3.18%)	7 (4.17%)	10 (6.41%)	13 (7.47%)	
	Balloon + stent	211 (32.21%)	50 (31.85%)	50 (29.76%)	54 (34.62%)	57 (32.76%)	
	Thrombolysis + stent	1 (0.15%)	1 (0.64%)	0 (0.00%)	0 (0.00%)	0 (0.00%)	
	All three	1 (0.15%)	1 (0.64%)	0 (0.00%)	0 (0.00%)	0 (0.00%)	
LVDS (mm)		40.00 (33.00–45.00)	40.00 (33.00–47.00)	40.00 (35.00–45.00)	39.50 (31.75–45.00)	39.00 (34.00–44.75)	0.984
LVEDD (mm)		53.00 (49.00–58.00)	56.00 (49.00–61.00)	53.00 (49.00–57.00)	53.00 (48.00–58.00)	53.00 (49.00–57.00)	0.413
LVEF (%)		39.00 (32.80–44.00)	39.00 (32.00–44.70)	38.00 (32.60–45.00)	39.00 (33.00–45.00)	39.00 (33.25–42.75)	0.846
In hospital malignant arrhythmias, n (%)		67 (10.23%)	9 (5.73%)	14 (8.33%)	20 (12.82%)	24 (13.79%)	0.044

Data are presented as Median (Quartile 1, Quartile 3) or n (%). Abbreviations: TyG, triglyceride glucose index; BMI, body mass index; SBP, systolic blood pressure; DBP, diastolic blood pressure; HbA1c, glycosylated hemoglobin, type A1C; eGFR, estimated glomerular filtration Rate; BNP, B type natriuretic peptide; TC, serum total cholesterol; LDL-C, low density lipoprotein cholesterol; HDL-C, high density lipoprotein cholesterol; CRP, C reactive protein; NE, neutrophil; WBC, white blood cell; LVDS, left ventricular end systolic dimension; LVEDD, left ventricular end diastolic diameter; LVEF, left ventricular ejection fraction; ACEI, angiotensin-converting enzyme inhibitor; ARB, angiotensin II receptor blocker; PCI, percutaneous coronary intervention.

### 3.2 Relationships of TyG Index Concentration With In-Hospital Malignant Arrhythmias

Throughout the median follow-up period of 8 days (6.00, 14.00), 67 (10.23%) patients showed in-hospital malignant arrhythmias. Cox regression analysis (Table 2) showed that higher TyG index levels were significantly linked to an elevated risk of in-hospital malignant arrhythmias (HR = 1.57, 95% CI: 1.06–2.32). In the quartile analysis, patients in Q3 (HR = 2.87, 95% CI: 1.22–6.75) and Q4 (HR = 2.99, 95% CI: 1.26–7.13) had markedly higher risks of malignant arrhythmias compared with those in Q1.

**Table 2.** **COX regression results for the association of changes in TyG and continuous TyG with Malignant arrhythmia in hospital among individuals aged ≥60 years with Ventricular aneurysm and diabetes**.

	Model I		Model II		Model III	
	HR (95% CI)	*p* value	HR (95% CI)	*p* value	HR (95% CI)	*p* value
Continuous	1.50 (1.07, 2.10)	0.018	1.48 (1.04, 2.13)	0.032	1.57 (1.06, 2.32)	0.024
Q1	Reference		Reference		Reference	
Q2	1.41 (0.61, 3.27)	0.418	1.50 (0.64, 3.51)	0.347	1.58 (0.65, 3.83)	0.309
Q3	2.31 (1.05, 5.08)	0.037	2.42 (1.07, 5.45)	0.034	2.87 (1.22, 6.75)	0.016
Q4	2.52 (1.17, 5.43)	0.018	2.65 (1.17, 5.98)	0.019	2.99 (1.26, 7.13)	0.013
*p* for trend		0.007		0.009		0.004

Model Ⅰ: crude model. Model Ⅱ: adjusted for Age, Gender, BMI, Smoke, Alcohol consumption. Model Ⅲ: adjusted for Age, Gender, BMI, Smoke, Alcohol consumption, SBP, DBP, eGFR, HbA1c, BNP, TC, TG, LDL-C, HDL-C, Hypertension, Hyperlipidemia, ACEI/ARB, Lipid lowering Drug, Aldosterone antagonist, Beta-blocker, Antiarrhythmic drugs, PCI status, LVDS, LVEDD, LVEF. Abbreviations: HR, hazard ratio; CI, confidence interval.

**Fig. 2.**
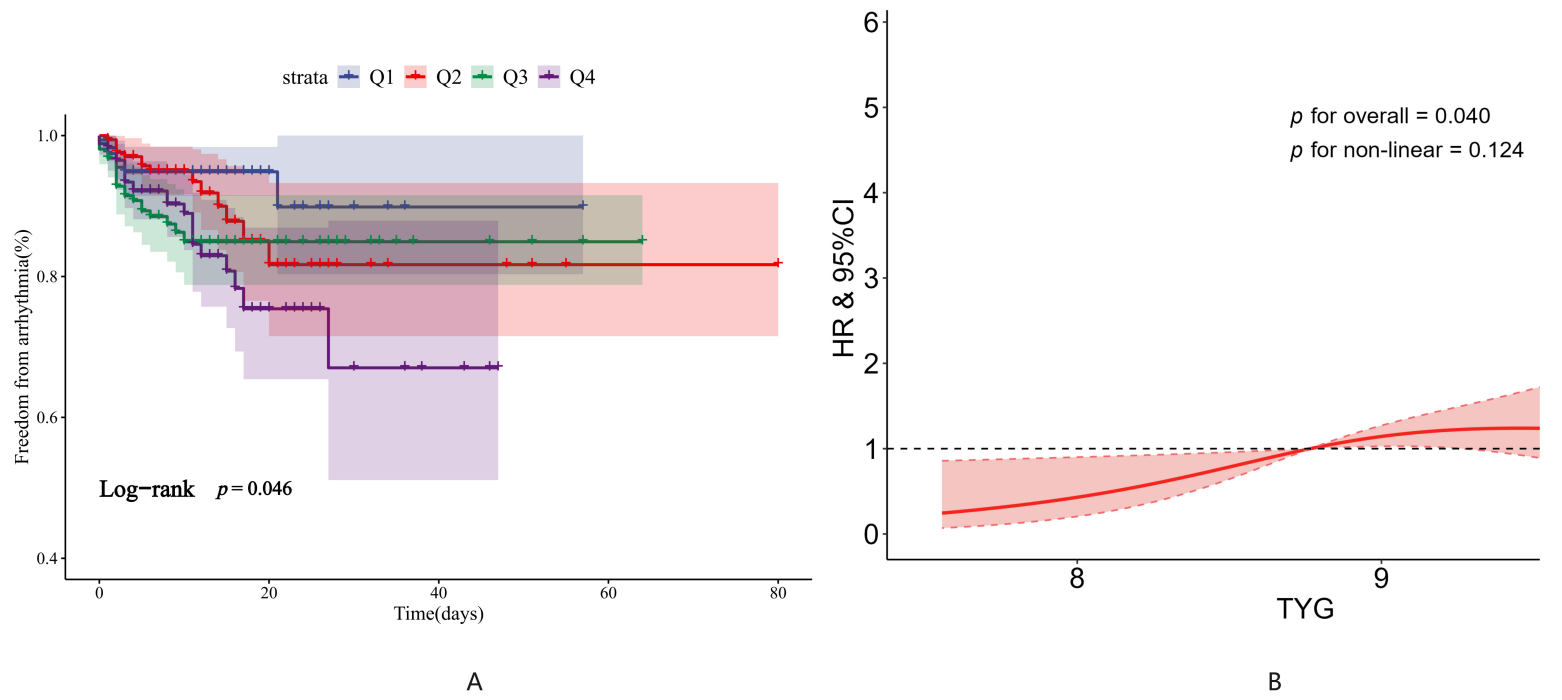
**The relationship between TyG and in-hospital malignant arrhythmias**. (A) Kaplan-Meier survival curves for in-hospital malignant arrhythmias according to TyG quartiles. (B) Restricted cubic spline regression analysis illustrating the association between the TyG index and in-hospital malignant arrhythmias.

The Kaplan-Meier (K-M) survival curves (Fig. 2A) showed that the incidence of malignant arrhythmias was markedly elevated in the Q4 group relative to Q1. Building on this gradient across TyG quartiles, Kaplan-Meier curves based on the ROC-derived exploratory cutoff (**Supplementary Fig. 2**) further demonstrated that patients in the high-TyG group experienced an elevated risk of in-hospital malignant arrhythmias compared to those with lower TyG levels. This exploratory cutoff serves as a preliminary reference for identifying high-risk participants, and requires further external validation before clinical application. In addition, the RCS model revealed a clear linear increase in the risk of in-hospital malignant arrhythmias with rising TyG levels (Fig. 2B).

### 3.3 Exploratory Pathway Analysis of TyG With In-Hospital Malignant Arrhythmias

**Fig. 3.**
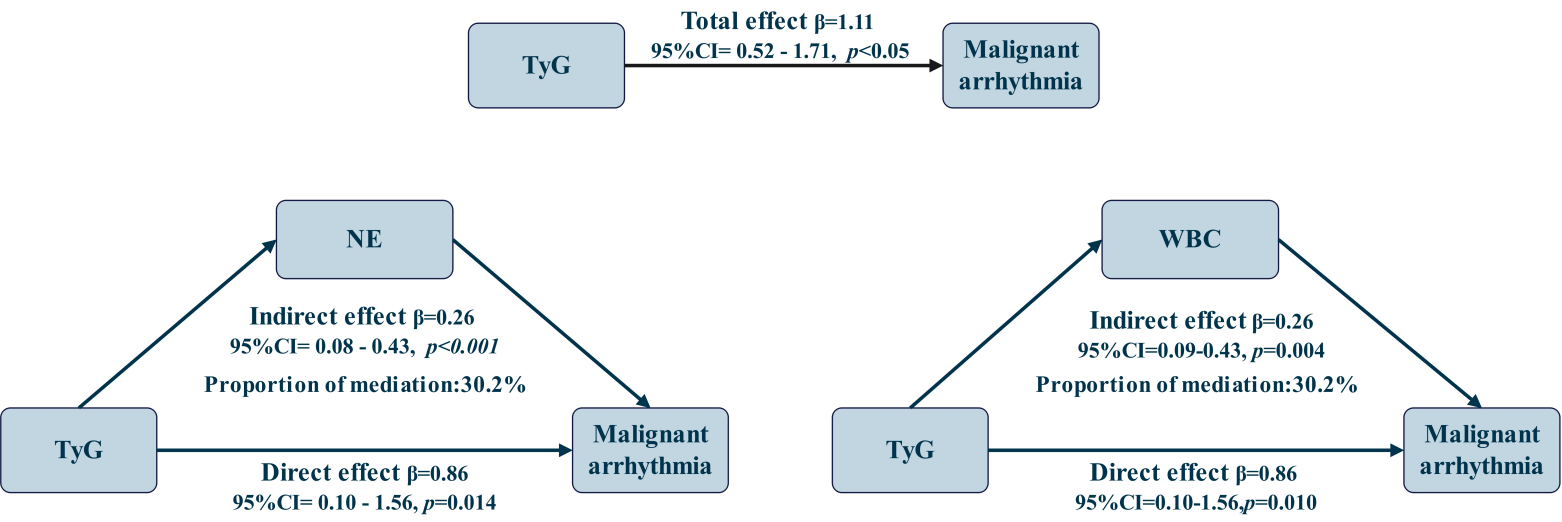
**Exploratory pathway analysis of the association between the TyG index and in-hospital malignant arrhythmias involving NE and WBC**.

The exploratory pathway analysis demonstrated a significant relationship between the TyG index and the risk of in-hospital malignant arrhythmias, with an overall association of β = 1.11 (95% CI: 0.52–1.71). In the single-pathway models, both neutrophil count and white blood cell count statistically explained a significant proportion of this relationship. The pathway involving NE was β = 0.26 (95% CI: 0.08–0.43), accounting for 30.2% of the overall association, whereas WBC showed a similar statistical pathway of β = 0.26 (95% CI: 0.09–0.43), also explaining 30.2% of the overall association (Fig. 3). These findings indicate that inflammation, reflected by NE and WBC, partially accounts for the association between TyG and malignant arrhythmias.

Considering the biological correlation and potential overlap between NE and WBC, a multiple-pathway model incorporating both markers was further constructed. The independent pathway associations of NE and WBC were diminished and no longer significant, while the combined pathway association remained significant (**Supplementary Table 3**). This pattern suggests partial redundancy between the two markers and supports inflammation as a shared underlying pathway.

To further characterize inflammatory mechanisms, we conducted additional analyses focusing on the composite inflammatory index SIRI. We found that SIRI exhibited a statistically significant pathway association (β = 0.09, 95% CI: 0.00–0.20), accounting for 8.5% of the overall association, further supporting a potential role of systemic inflammation in this link (**Supplementary Fig. 3**).

### 3.4 Subgroup Analyses

**Fig. 4.**
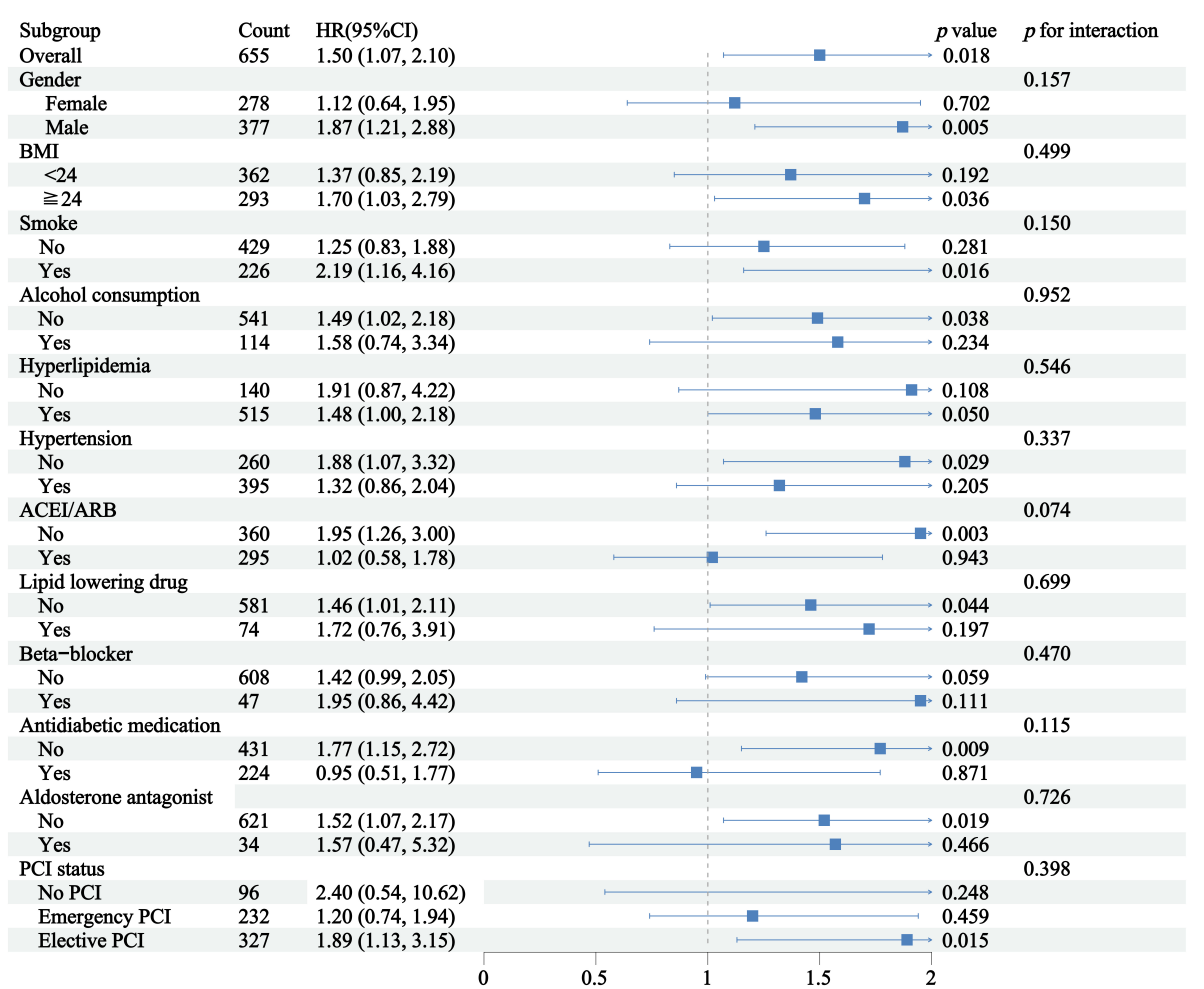
**Subgroup analyses of TyG index and in-hospital malignant arrhythmia in elderly diabetic patients with ventricular aneurysm**.

Given the complex medication regimen of these patients, subgroup analyses were performed to verify the consistency of TyG’s predictive value across different therapeutic backgrounds. Subgroup analyses revealed that the relationship linking the TyG index and in-hospital malignant arrhythmias was consistent across all examined strata, including gender, BMI, smoke, alcohol consumption, hyperlipidemia, hypertension, ACEI/ARB therapy, lipid-lowering drugs, Beta-blocker use, antidiabetic medication, aldosterone antagonist use, and PCI status, with no significant interactions observed (Fig. 4; all *p* for interaction > 0.05).

## 4. Discussion

The findings of this study indicate that elevated TyG levels are strongly linked to a higher risk of in-hospital malignant arrhythmias among elderly patients with LVA and diabetes mellitus. Our findings showed that elevated TyG index levels were independently associated with a higher risk of malignant arrhythmias; individuals in the highest quartile (Q4) exhibited a nearly threefold increased risk compared to those in the lowest quartile (Q1). This finding aligns with prior research demonstrating that IR, as reflected by the TyG index, contributes to adverse cardiovascular outcomes, including arrhythmias [20]. The acute arrhythmic burden in AMI patients with new-onset LVA is highlighted by the observed in-hospital incidence of malignant arrhythmias (10.23%) in our cohort. This number is consistent with research in ventricular dysfunction (roughly 6%) [2], despite being lower than rates in some unselected AMI populations. This suggestion opportunity for focused interventions like improved monitoring or preventive treatments. Longer-term follow-up studies in the future could investigate whether this risk persists after hospitalization.

Triglycerides represent the main form in which the body stores fat, consisting of three fatty acid chains attached to a glycerol backbone [21]. Elevated levels of triglycerides, known as hypertriglyceridemia, have been linked to a heightened susceptibility to cardiovascular disorders [22]. By combining triglyceride and glucose measurements, this metric offers a clinically reliable means of estimating susceptibility to cardiovascular disease [20]. The association between the TyG index and malignant arrhythmias can be illustrated by several pathophysiological mechanisms. The TyG index has been implicated in promoting myocardial fibrosis and oxidative stress, both of which are crucial in the development of arrhythmias [23, 24]. Myocardial fibrosis can cause structural remodeling of the ventricles, forming a substrate for reentrant arrhythmias, while oxidative stress can impair the function of ion channels and gap junctions, thereby disrupting the normal electrical conduction system of the heart [25, 26]. The exploratory pathway analysis was conducted to elucidate potential biological pathways linking metabolic insulin resistance to electrophysiological instability, indicating that NE, WBC, and SIRI partially account for the association between the TyG index and malignant arrhythmias [19, 20, 27, 28].

Growing evidence suggests that IR and chronic inflammation function as mutually reinforcing components of a broader immune-metabolic disorder [29]. Persistent low-grade inflammation can exacerbate metabolic dysfunction, while impaired insulin signaling further amplifies systemic inflammatory activity, together creating an internal environment conducive to cardiovascular injury [30, 31]. These immune-metabolic disturbances promote structural remodeling, oxidative stress, and electrical instability of the myocardium, thereby increasing susceptibility to malignant arrhythmias [32].

At the same time, composite inflammatory indices such as the SIRI have emerged as reliable markers of systemic inflammatory burden and have been shown to predict cardiovascular events and atherosclerotic progression in large cohort studies [33]. Consistent with these findings, our exploratory pathway analysis demonstrated that inflammatory indices partially accounted for the association between the TyG index and in-hospital malignant arrhythmias, supporting the role of inflammation as a potential mechanistic bridge linking metabolic dysregulation to arrhythmic risk. It is worth noting that the proportion of the association statistically explained by SIRI was relatively small. This suggests that the relationship between TyG and malignant arrhythmias is likely multifactorial, involving other unmeasured pathways. Therefore, this finding should be interpreted as a partial statistical explanation, and further research is warranted to explore additional underlying mechanisms in the future.

This study identifies an exploratory TyG cutoff value of 9.01. While it requires further external validation before being considered a clinically actionable tool for risk stratification, this threshold provides a preliminary reference for identifying high-risk individuals. Given the substantial morbidity and mortality linked to malignant arrhythmias in elderly patients with LVA, early recognition and management of high-risk individuals are essential for improving clinical outcomes. The simplicity and cost-effectiveness of the TyG index make it an attractive option for clinical application. In clinical practice, the TyG index can be readily incorporated into routine assessments to facilitate early screening of arrhythmias. The calculation of the TyG index relies solely on routine biochemical measurements, specifically fasting triglyceride and glucose levels, which are commonly obtained during routine blood examination [34]. Therefore, it doesn’t need additional specialized equipment or complex procedures. Once the TyG index is measured and individuals are identified as high-risk based on the cutoff value of 9.01, a range of specific interventions can be applied to reduce the risk of arrhythmias. Concurrently, lifestyle modifications are equally important and include dietary modification, increased physical activity, weight control, and smoking cessation [35].

The RCS analysis indicated that the relationship between the TyG index and the risk of in-hospital malignant arrhythmias followed a linear relationship. This linear relationship suggests that even small increases in the TyG index are associated with a proportional increase in the risk of malignant arrhythmias, highlighting the importance of early detection and management of insulin resistance in this high-risk population.

The subgroup analyses showed none of the examined clinical indices significantly modified the association of TyG levels with in-hospital malignant arrhythmias, suggesting that the effect of TyG on arrhythmic risk in elderly patients with LVA is largely independent of these common clinical factors. Such consistent findings across subgroups support the strength of TyG as a dependable indicator of metabolic risk. The result enhances the potential applicability of TyG in risk stratification and supports its integration into routine clinical assessment for early identification of high-risk individuals.

### Limitations

Despite its strengths, this study has several limitations. First, the retrospective design and the exclusion of cases with missing earlier records may have introduced a degree of selection bias. Second, the relatively short observation window precludes the evaluation of long-term prognostic implications. Third, the lack of temporal separation between the assessment of the TyG index and inflammatory markers restricts our exploratory pathway analysis to being hypothesis-generating, precluding any direct causal inferences. In addition, the cohort was limited to elderly patients with LVA and diabetes mellitus, which may constrain the generalizability of the results. Therefore, external validation in larger and more diverse populations is needed. Future prospective studies with longer follow-up periods will be essential to validate these findings and further delineate the temporal associations underlying these mechanisms.

## 5. Conclusions

In summary, this research demonstrates that the TyG index is independently associated within-hospital malignant arrhythmias among elderly patients with LVA and diabetes. Since it is an accessible and practical surrogate of IR, the TyG index may offer a valuable preliminary markerfor early risk assessment and the implementation of more targeted clinical management.

## Availability of Data and Materials

The dataset examined in this study is available upon reasonable request from the corresponding author.
