# Supplementary material for: Association of Triglyceride-Glucose Index With In-Hospital Malignant Arrhythmias in Older Patients With Diabetes Mellitus and Left Ventricular Aneurysm: A Retrospective Study
Source: Rev Cardiovasc Med. 2026 May 18;27(5):47761. doi: 10.31083/RCM47761 (PMC13227363; doi:10.31083/RCM47761)
Supplement: Supplementary file 1 [file 2153-8174-27-5-47761-s1.zip › Supplementary Material.docx]

**SUPPLEMENTAL INFORMATION**

**Supplementary Table 1:** Collinearity diagnostics among the variables.

**Supplementary Table 2:** Baseline clinical characteristics of patients stratified by the optimal cutoff point of the TyG index

**Supplementary Table 3.** Multiple-mediator analysis of the association between the TyG index and in-hospital malignant arrhythmias.

**Supplementary Fig. 1.** ROC Curve and Best Threshold Value of the Triglyceride-Glucose Index for Predicting In-Hospital Malignant Arrhythmias

**Supplementary Fig. 2.** Kaplan-Meier survival curves for in-hospital malignant arrhythmias according to TYG index cut-off values.

**Supplementary Fig. 3.** Exploratory pathway analysis of the association between the TyG index and in-hospital malignant arrhythmias involving SIRI.

**Supplementary Table 1:** Collinearity diagnostics among the variables.

| Variable | GVIF | Df | GVIF^(1/(2*Df)) |
| --- | --- | --- | --- |
| TyG | 1.37 | 1 | 1.17 |
| Age | 1.30 | 1 | 1.14 |
| Gender | 1.78 | 1 | 1.33 |
| Smoke | 1.91 | 1 | 1.38 |
| Alcohol consumption | 1.45 | 1 | 1.20 |
| SBP | 1.83 | 1 | 1.35 |
| DBP | 1.94 | 1 | 1.39 |
| BMI | 1.09 | 1 | 1.04 |
| TC | 1.12 | 1 | 1.06 |
| LDL-C | 1.32 | 1 | 1.15 |
| HDL-C | 1.78 | 1 | 1.33 |
| eGFR | 1.67 | 1 | 1.29 |
| BNP | 1.80 | 1 | 1.34 |
| Hyperlipidemia | 1.65 | 1 | 1.28 |
| Hypertension | 1.20 | 1 | 1.10 |
| ACEI/ARB | 1.31 | 1 | 1.14 |
| Lipid lowering drug | 2.46 | 1 | 1.57 |
| Beta-blocker | 2.40 | 1 | 1.55 |
| Aldosterone antagonist | 1.64 | 1 | 1.28 |
| Antiarrhythmic drugs | 1.15 | 1 | 1.07 |
| LVDS | 3.06 | 1 | 1.75 |
| LVEDD | 2.99 | 1 | 1.73 |
| LVEF | 1.16 | 1 | 1.08 |
| PCI status | 1.21 | 2 | 1.05 |

Abbreviations: **GVIF**, **Generalized Variance Inflation Factor; Df**: **Degrees of Freedom;** TYG, Triglyceride Glucose Index; BMI, Body mass index; SBP, systolic blood pressure; DBP, diastolic blood pressure; eGFR, Estimated Glomerular Filtration Rate; BNP, B-type Natriuretic Peptide; TC, Serum Total Cholesterol; LDL-C, Low-density lipoprotein cholesterol; HDL-C, High-density lipoprotein cholesterol; ACEI, angiotensin-converting enzyme inhibitor; ARB, Angiotensin II receptor blocker; PCI, percutaneous coronary intervention; LVDS, Left Ventricular End-Systolic Dimension; LVEDD, Left Ventricular End-Diastolic Diameter; LVEF, Left Ventricular Ejection Fraction.

**Supplementary Table 2:** Baseline clinical characteristics of patients stratified by the optimal cutoff point of the TyG index

| Variables | Overall | LOW TyG (N = 388) | HIGH TyG (N = 267) | p value |
| --- | --- | --- | --- | --- |
| TyG | 8.82 (8.42-9.33) | 8.51 (8.19-8.74) | 9.45 (9.22-9.80) | <0.001 |
| Age | 75.00 (68.00-79.00) | 75.50 (70.00-80.00) | 73.00 (66.50-78.00) | <0.001 |
| Males, n (%) | 377 (57.56%) | 258 (66.49%) | 119 (44.57%) | <0.001 |
| BMI | 23.54 (21.64, 25.71) | 23.14 (20.96, 25.67) | 23.78 (22.05, 25.71) | 0.061 |
| Smoke, n (%) | 226 (34.50%) | 159 (40.98%) | 67 (25.09%) | <0.001 |
| Alcohol consumption,n (%) | 114 (17.40%) | 72 (18.56%) | 42 (15.73%) | 0.405 |
| SBP (mmHg) | 115.00 (103.00-130.00) | 114.00 (103.00-128.25) | 116.00 (103.50-131.50) | 0.152 |
| DBP (mmHg) | 68.00 (61.00-75.00) | 67.00 (61.00-74.00) | 68.00 (61.00-75.50) | 0.135 |
| eGFR | 66.40 (45.40-85.90) | 67.70 (50.13-84.50) | 64.60 (39.00-87.95) | 0.165 |
| HbA1c (%) | 7.30 (6.40-8.40) | 7.00 (6.20-8.00) | 7.60 (6.70-9.10) | <0.001 |
| BNP (mmol/L) | 852.00 (384.00-1850.50) | 840.00 (348.50-1681.00) | 925.00 (446.00-1925.50) | 0.422 |
| TC (mmol/L) | 2.72 (0.74-4.27) | 2.81 (0.64-4.16) | 2.42 (0.89-4.56) | 0.075 |
| TG (mmol/L) | 1.22 (0.92-1.69) | 1.00 (0.80-1.24) | 1.75 (1.37-2.20) | <0.001 |
| LDL-C ( mol/L) | 2.59 (2.05-3.23) | 2.50 (1.90-3.06) | 2.78 (2.17-3.53) | <0.001 |
| HDL-C (mmol/L) | 0.98 (0.80-1.17) | 0.99 (0.82-1.17) | 0.95 (0.78-1.17) | 0.578 |
| CRP (mg/L) | 26.20 (9.90-65.55) | 24.60 (8.18-62.13) | 31.40 (12.45-71.10) | 0.002 |
| WBC (×10^9/L) | 8.37 (6.44-11.13) | 7.80 (5.92-10.09) | 9.70 (7.39-12.20) | <0.001 |
| NE (×10^9/L) | 6.00 (4.09-8.88) | 5.43 (3.72-7.72) | 7.20 (5.00-9.98) | <0.001 |
| Lymphocyte (×10^9/L) | 1.40 (1.02-1.80) | 1.36 (1.00-1.77) | 1.41 (1.10-1.85) | 0.011 |
| Monocytes (×10^9/L) | 0.60 (0.43-0.88) | 0.60 (0.40-0.80) | 0.64 (0.48-0.98) | 0.003 |
| Hyperlipidemia, n (%) | 515 (78.63%) | 279 (71.91%) | 236 (88.39%) | <0.001 |
| Hypertension, n (%) | 395 (60.31%) | 211 (54.38%) | 184 (68.91%) | <0.001 |
| ACEI/ARB, n (%) | 295 (45.04%) | 177 (45.62%) | 118 (44.19%) | 0.779 |
| Lipid lowering drug, n (%) | 74 (11.30%) | 46 (11.86%) | 28 (10.49%) | 0.676 |
| Antidiabetic medication, n (%) | 224 (34.20%) | 94 (24.23%) | 130 (48.69%) | <0.001 |
| Beta-blocker, n (%) | 47 (7.18%) | 33 (8.51%) | 14 (5.24%) | 0.151 |
| In-hospital beta-blocker use, n (%) | 375 (57.25%) | 205 (52.84%) | 170 (63.67%) | 0.007 |
| Aldosterone antagonist, n (%) | 34 (5.19%) | 20 (5.15%) | 14 (5.24%) | 1.000 |
| In-hospital aldosterone antagonist use, n (%) | 365 (55.73%) | 210 (54.12%) | 155 (58.05%) | 0.360 |
| Antiarrhythmic drugs, n (%) | 28 (4.27%) | 18 (4.64%) | 10 (3.75%) | 0.719 |
| In-hospital antiarrhythmic drug use, n (%) | 79 (12.06%) | 45 (11.60%) | 34 (12.73%) | 0.752 |
| PCI status, n (%) |  |  |  | 0.850 |
| No PCI | 96 (14.66%) | 55 (14.18%) | 41 (15.36%) |  |
| Emergency PCI | 232 (35.42%) | 136 (35.05%) | 96 (35.96%) |  |
| Elective PCI | 327 (49.92%) | 197 (50.77%) | 130 (48.69%) |  |
| PCI type, n (%) |  |  |  |  |
| No PCI | 96 (14.66%) | 55 (14.18%) | 41 (15.36%) |  |
| Conservative strategy | 110 (16.79%) | 64 (16.49%) | 46 (17.23%) |  |
| Stent only | 201 (30.69%) | 127 (32.73%) | 74 (27.72%) |  |
| Balloon only | 35 (5.34%) | 16 (4.12%) | 19 (7.12%) |  |
| Balloon + stent | 211 (32.21%) | 124 (31.96%) | 87 (32.58%) |  |
| Thrombolysis + stent | 1 (0.15%) | 1 (0.26%) | 0 (0.00%) |  |
| All three | 1 (0.15%) | 1 (0.26%) | 0 (0.00%) |  |
| LVDS (mm) | 40.00 (33.00-45.00) | 40.00 (33.00-45.00) | 39.00 (33.00-45.00) | 0.500 |
| LVEDD (mm) | 53.00 (49.00-58.00) | 53.00 (49.00-59.00) | 53.00 (49.00-58.00) | 0.552 |
| LVEF (%) | 39.00 (32.80-44.00) | 38.50 (32.00-45.00) | 39.00 (33.50-43.65) | 0.277 |
| In hospital malignant arrhythmias, n (%) | 67 (10.23%) | 30 (7.73%) | 37 (13.86%) | 0.016 |

Data are presented as Median (Quartile 1, Quartile 3) or n (%)

Abbreviations: TYG, Triglyceride Glucose Index; BMI, Body mass index; SBP, systolic blood pressure; DBP, diastolic blood pressure; HbA1c, Glycosylated Hemoglobin, Type A1C; eGFR, Estimated Glomerular Filtration Rate; BNP, B type Natriuretic Peptide; TC, Serum Total Cholesterol; TG, Triglycerides; LDL-C, Low density lipoprotein cholesterol; HDL-C, High density lipoprotein cholesterol; CRP, C reactive protein; NE, neutrophil; WBC, white blood cell; LVDS, Left Ventricular End Systolic Dimension; LVEDD: Left Ventricular End Diastolic Diameter; LVEF: Left Ventricular Ejection Fraction; ACEI, angiotensin II coenzyme inhibitor; ARB, angiotensin II receptor blocker; PCI, percutaneous coronary intervention.

**Supplementary Table 3.** Multiple-mediator analysis of the association between the TyG index and in-hospital malignant arrhythmias.

| **Pathway** | **Estimate (log HR)** | **95% CI** | ***p* value** |
| --- | --- | --- | --- |
| Indirect via NE | 0.69 | -0.05 – 1.32 | 0.062 |
| Indirect via WBC | -0.40 | -1.03 – 0.20 | 0.200 |
| Total indirect effect | 0.29 | 0.10 – 0.46 | <0.001 |
| Direct effect (c′) | 0.90 | 0.01 – 1.54 | 0.018 |

**
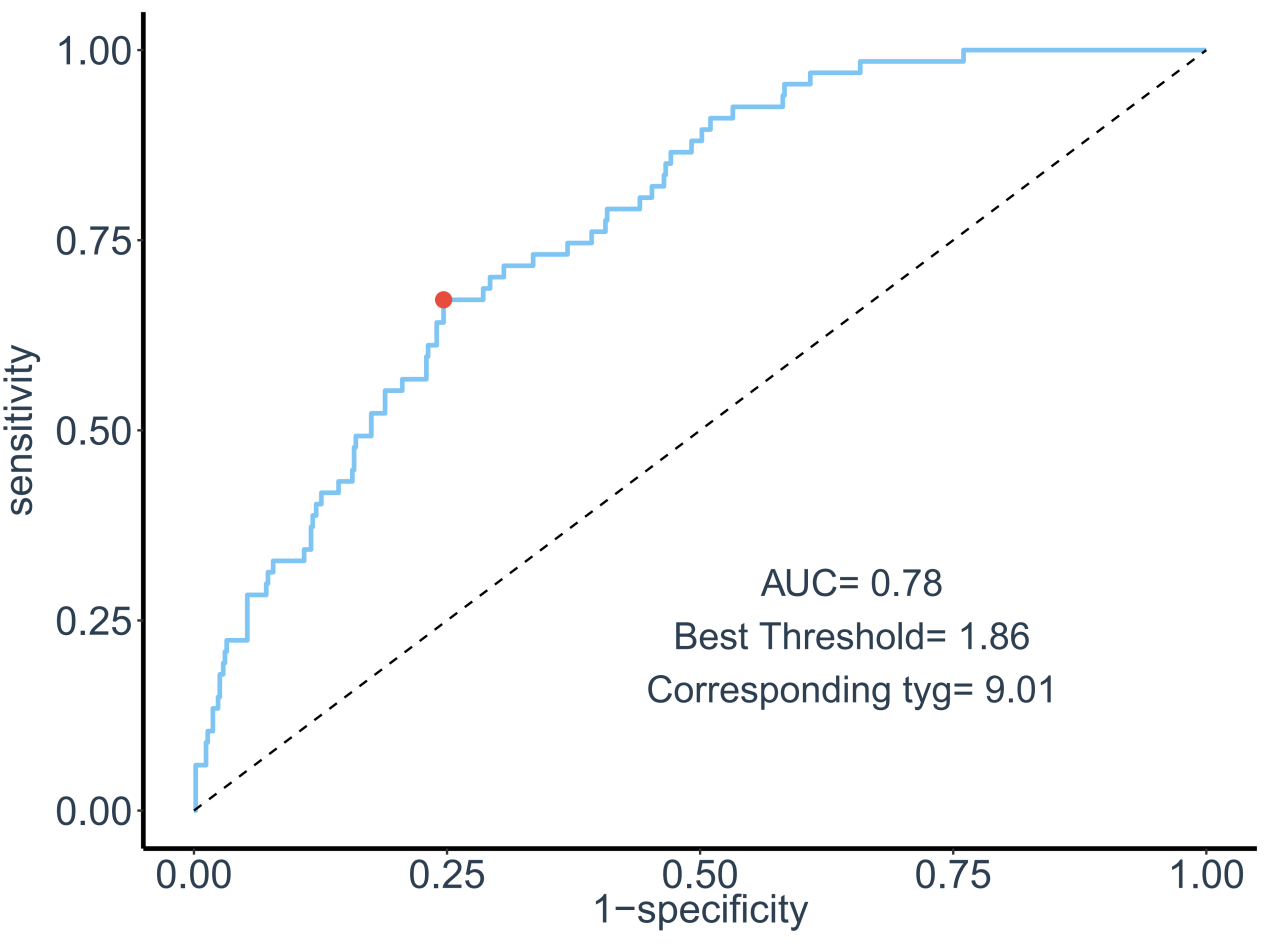
**

**Supplementary Fig. 1.** ROC Curve and Best Threshold Value of the Triglyceride-Glucose Index for Predicting In-Hospital Malignant Arrhythmias

**
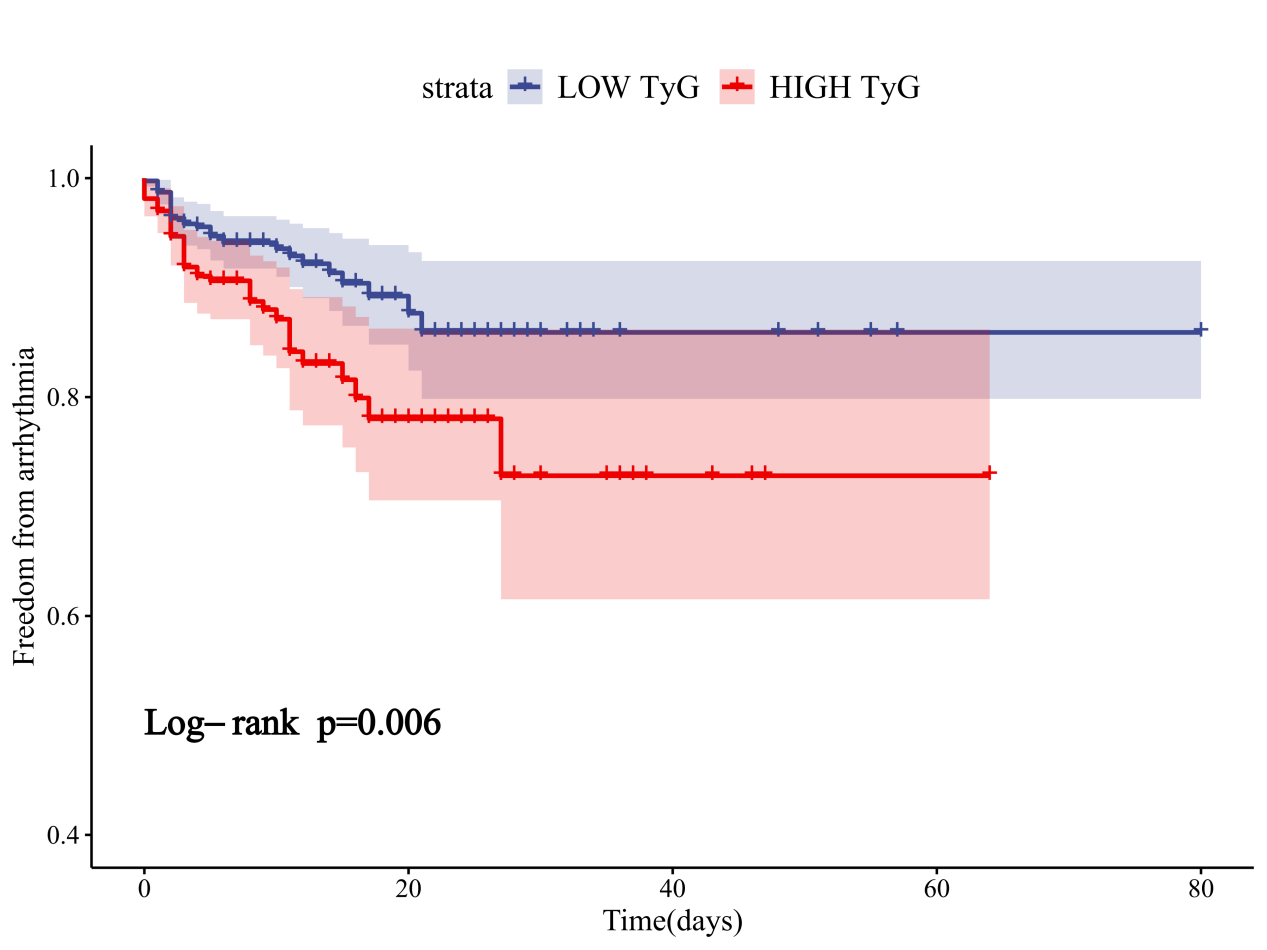
**

**Supplementary Fig. 2.** Kaplan-Meier survival curves for in-hospital malignant arrhythmias according to TYG index cut-off values.


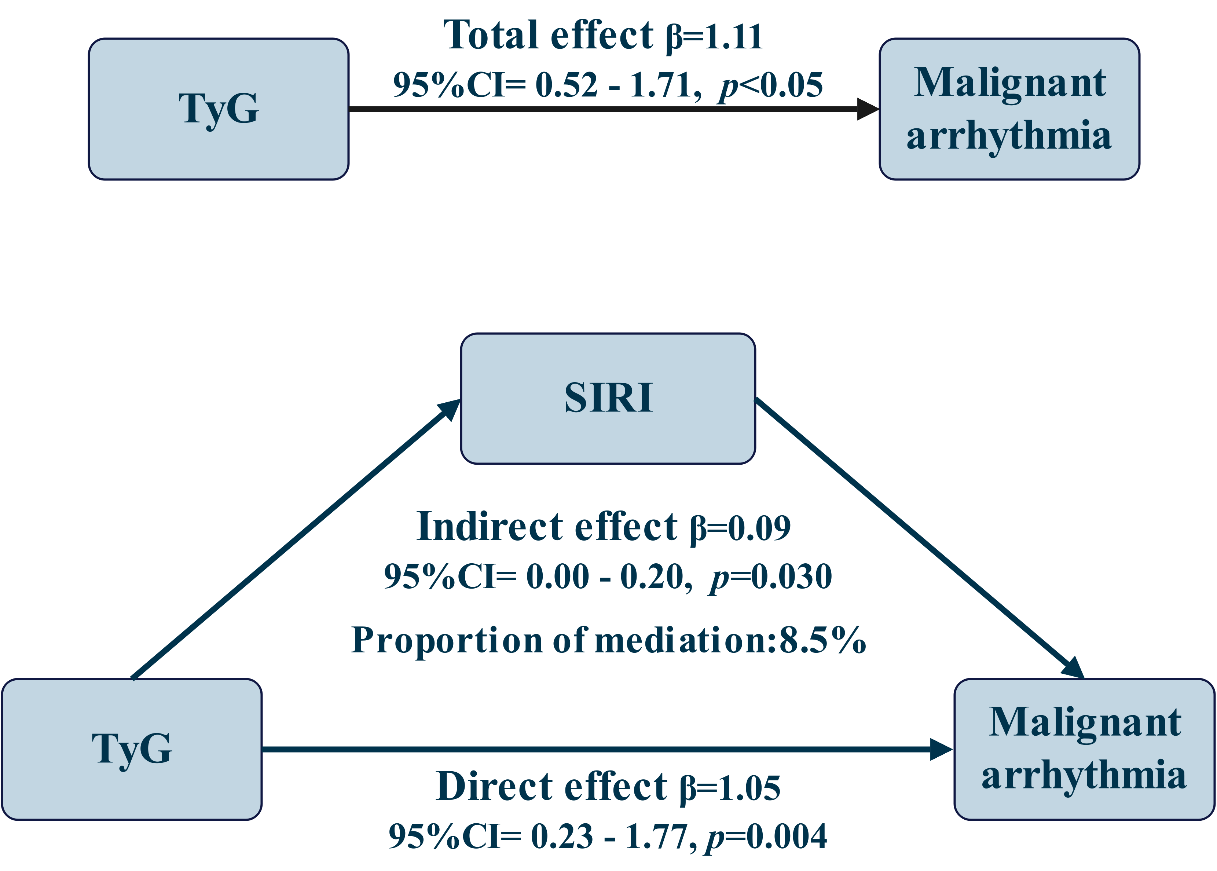


**Supplementary Fig. 3.** Exploratory pathway analysis of the association between the TyG index and in-hospital malignant arrhythmias involving SIRI.
